# College, Hospital and Dispensary Notes

**Published:** 1897-08

**Authors:** 


### College, Hospital and Dispensary Notes.

THE Buffalo German Hospital appears to be an assured success. At a meeting of the directors held June 4, 1897, Jacob Lang and Edwin G. S. Miller, on behalf of the children and estate of the late Gerhard Lang, tendered to them the sum of $5,000, to be used toward building the fine structure proposed for the hospital.

The gift was accepted promptly, and a resolution was adopted expressing the gratitude of the officers for the contribution, and directing that some suitable testimonial be placed in the new building to commemorate the generosity.

The persons participating in the contribution are Jacob, Catherine, Emma A. M., Bertha, Mary M., and Ludewina Lang, Anna E. and Edwin G. S. Miller, children, executors and executrices. A copy of the resolution was forwarded to each of these persons.

The meeting of the directors was held for the purpose of selecting a site for the new building. The property on the west side of Jefferson street, 200 feet south of Genesee street, which has a frontage of 150 feet and depth of 192 feet, and which has been under option for some time, was accepted and a committee was appointed to make the purchase and negotiate a loan sufficient to defray the expense of erecting the building.

It is estimated that the building will cost about $25,000 and the land about $10,000.

The board selected Geo. J. Metzger as architect for the building, he having manifested considerable interest in the project. He agreed to have plans ready by July 1st, and an effort will be made to have the building completed before the snow flies next winter.

The work of removing the old buildings on the site has already begun.

The directors of the hospital are Chas. J. North, Dr. Charles H. W. Auel, Jacob J. Lang, Ottomar Reinecke, Edwin G. S. Miller, William Simon, Jacob Stern and Charles Duchman. They all feel much gratified over the gift and the progress of the enterprise.

At a meeting of the board of trustees of the University of Cincinnati, held Saturday, May 15, 1897, a number of important questions were brought up, among which was the movement looking toward legislation by which all applicants for matriculation in the medical schools of the state shall have to pass an examination. The *Enquirer* is authority for the following report of the meeting, as it related to this subject:

The trustees and the faculties of the University of Cincinnati met for the first time in joint session. The meeting was called at the suggestion of Dr. C. A. L. Reed, of the university trustees. President Jones, of the board of trustees, was unable to be present, and in his absence Dr. Reamy was chosen to preside. Dr. Reed was the first to take the floor and, in an exhaustive paper, laid before the meeting a number of topics for its consideration. The most important matter, and the one which was discussed at great length, was that of an examination which shall be the same all over the state for admission to professional schools—that is, schools from which lawyers and doctors are graduated. A movement looking towards legislation by which all applicants for matriculation in these schools shall have to pass an examination by a state board has been started by the Columbus Academy of Medicine, and a number of other medical colleges have indorsed the movement. It was to take action on this matter that the meeting was called, but no action was taken, the matter being laid on the table for one year.—*Columbus Med. Jour.*

---

Dr. Louise Downer, formerly of the Warsaw salt baths, announces to the medical profession that she has opened a sanitarium at 174 N. Pearl street, Buffalo, N. Y. She has with her an able masseuse, Miss Abigail Hickey, favorably known by people of this city and highly recommended. The line of treatment will consist of brine, stimulating, sulphur, electric and cabinet baths, salt sponges and rubs, oil and alcohol massage. She will be pleased to follow any line of treatment that the attending physician may suggest. It is desirable that application be made in advance. Physicians are cordially invited to call. Telephone, Bryant 670.

---

A hospital for the treatment of consumptives and persons suffering with contagious diseases is contemplated at Asheville, N. C. Mr. George W. Vanderbilt, whose generosity has made him so justly popular, proposes to donate $100,000 toward the construction of the institution, which will be, when completed, one of the finest hospitals in the south. Mr. Vanderbilt's philanthropy is of the right sort, and if more wealthy men would build or endow hospitals, they would leave an enduring memory of the most savory nature.

